# Backward, upside down and inside out — the regulatory evolution of vertebrates

**DOI:** 10.1093/nsr/nwz067

**Published:** 2019-05-23

**Authors:** Chung-I Wu, Anlong Xu

**Affiliations:** 1 School of Life Science, Sun Yat-sen University, China; 2 Department of Ecology and Evolution, University of Chicago, USA *Corresponding authors. E-mails: wzhongyi@mail.sysu.edu.cn; lssxal@mail.sysu.edu.cn

In this issue of *NSR*, the research team led by Jiang Liu of Beijing Institute of Genomics, publishes a paper [1] on the evolutionary transition between invertebrates and vertebrates.

For geneticists, especially the modern brand to whom the detailed molecular mechanism is the only reason to study biology, major evolutionary transitions would be the testing ground of that conviction. This is where vertebrates come in. Deuterostome appears to get the anterior–posterior axis backward (as far as protostomes see it). Étienne Geoffroy Saint-Hilaire forwarded the curious hypothesis that vertebrates are upside-down creatures compared with invertebrates. The endo- vs. exo-skeletal system is another distinguishing feature between them; we lump in the backbone even though it is unique in its own way. Finally, for a good measure, there is the adaptive immunity acquired at this evolutionary transition.

Is it then possible to understand, in molecular terms, anything about vertebrates that sets them apart from invertebrates? For specific features, there are answers. Saint-Hilaire's hypothesis has in fact acquired some support from modern molecular genetic studies [2,3]. For adaptive immunity, the V(D)J recombination of immunoglobulins has a fascinating origin connected to transposons [4,5]. In general, however, the gene-centric approach may not work. After all, this approach encounters a nearly insurmountable hurdle just trying to understand the genetic differences between sibling species that are identical in their morphology. Between them, hundreds of genes have diverged functionally in the spermatogenic program alone [6,7]. One could even argue that practically every gene plays a role in the divergence between congeneric species.

If the conundrum of the vertebrate–invertebrate transition is to be cracked in a general way, where might be the molecular entry point? Xu *et al.* show that DNA-methylation reprogramming is associated with the development, reproduction and adaptive immunity in vertebrates, but not in invertebrates. No less interesting, they show that the HOX cluster, which has been speculated to be the genic key to this transition, shows dynamic DNA-methylation patterns in vertebrates, but not in invertebrates.

Although this study does not provide specific molecular mechanistic answers, it is also dubious that the wide gaps in major evolutionary transitions can be bridged this way. Xu *et al.* at least offer an alternative, which may play a partial role in orchestrating the transition from invertebrates to vertebrates. Unlike other descriptions of the vertebrate–invertebrate transition, methylation *is* a regulatory mechanism of a rather complex kind in itself.
